# Histone acetyltransferase inhibitor CPTH6 preferentially targets lung cancer stem-like cells

**DOI:** 10.18632/oncotarget.7238

**Published:** 2016-02-08

**Authors:** Marta Di Martile, Marianna Desideri, Teresa De Luca, Chiara Gabellini, Simonetta Buglioni, Adriana Eramo, Giovanni Sette, Michele Milella, Dante Rotili, Antonello Mai, Simone Carradori, Daniela Secci, Ruggero De Maria, Donatella Del Bufalo, Daniela Trisciuoglio

**Affiliations:** ^1^ Department of Research, Advanced Diagnostics and Technological Innovation, Regina Elena National Cancer Institute, Rome, Italy; ^2^ Department of Hematology, Oncology and Molecular Medicine, Istituto Superiore di Sanità, Rome, Italy; ^3^ Clinical and Experimental Oncology Department, Regina Elena National Cancer Institute, Rome, Italy; ^4^ Department of Drug Chemistry and Technologies, ‘Sapienza’ University, Rome, Italy; ^5^ Pasteur Institute, Cenci Bolognetti Foundation, ‘Sapienza’ University, Rome, Italy; ^6^ Scientific Director, Regina Elena National Cancer Institute, Rome, Italy

**Keywords:** HAT inhibitors, cancer stem cells, acetylation, apoptosis, non-small cell lung cancer

## Abstract

Cancer stem cells (CSCs) play an important role in tumor initiation, progression, therapeutic failure and tumor relapse. In this study, we evaluated the efficacy of the thiazole derivative 3-methylcyclopentylidene-[4-(4′-chlorophenyl)thiazol-2-yl]hydrazone (CPTH6), a novel pCAF and Gcn5 histone acetyltransferase inhibitor, as a small molecule that preferentially targets lung cancer stem-like cells (LCSCs) derived from non-small cell lung cancer (NSCLC) patients. Notably, although CPTH6 inhibits the growth of both LCSC and NSCLC cell lines, LCSCs exhibit greater growth inhibition than established NSCLC cells. Growth inhibitory effect of CPTH6 in LCSC lines is primarily due to apoptosis induction. Of note, differentiated progeny of LCSC lines is more resistant to CPTH6 in terms of loss of cell viability and reduction of protein acetylation, when compared to their undifferentiated counterparts. Interestingly, in LCSC lines CPTH6 treatment is also associated with a reduction of stemness markers. By using different HAT inhibitors we provide clear evidence that inhibition of HAT confers a strong preferential inhibitory effect on cell viability of undifferentiated LCSC lines when compared to their differentiated progeny. *In vivo*, CPTH6 is able to inhibit the growth of LCSC-derived xenografts and to reduce cancer stem cell content in treated tumors, as evidenced by marked reduction of tumor-initiating capacity in limiting dilution assays. Strikingly, the ability of CPTH6 to inhibit tubulin acetylation is also confirmed *in vivo*. Overall, our studies propose histone acetyltransferase inhibition as an attractive target for cancer therapy of NSCLC.

## INTRODUCTION

Lung cancer is the leading cause of cancer-related deaths. Non-small cell lung cancer (NSCLC) accounts for ∼85% of all lung cancer cases [1]. The disease control achieved with classical chemotherapy doublets in advanced or metastatic NSCLC is usually restricted to only a few months [2]. Therefore, there is a need for development of novel agents that can be added to and improve the effect of traditional chemotherapy. In recent years, with growing insight into molecular alterations in lung cancer, tremendous efforts have been made to identify new anticancer agents. Aberrant epigenetic regulation is a frequent event in NSCLC, and both altered DNA CpG methylation and histone post-translational modifications have been shown to have both predictive and prognostic significance in this disease [3, 4]. Thus, it has been recently proposed that patients with NSCLC might benefit from treatment with epigenetic drugs [5]. Histone acetyltransferase inhibitors (HATi) belong to the family of epigenetic drugs and represent a heterogeneous group of compounds able to alter histone and non-histone protein functions [6]. Several molecules with HAT inhibitory activity have been identified and some of them showed to induce cell death preferentially in cancer cells when compared to normal ones [6–10]. Among them, the thiazole derivative 3-methyl-cyclopentylidene-[4-(4′-chlorophenyl)thiazol-2-yl)]hydrazone (CPTH6), has been characterized by our group as a novel Gcn5 and pCAF HAT inhibitor, able to activate the apoptotic program and to modulate the autophagic flux in a panel of tumor cell lines [11, 12]. Cancer stem cells (CSCs) have been proposed as potential culprits not only of the tumor initiation and progression, but also of therapeutic failure and tumor relapse, mainly because of their intrinsic resistance to conventional drugs [13]. Growing evidence shows a connection between epigenetic abnormalities in cancer and subpopulations with stem cells-like traits [14–16]. CSCs have been identified and characterized by the expression of markers, including CD133, CD44, aldehyde dehydrogenase (ALDH), side population [17–21], and by their cellular traits such as morphology, colony formation ability, and other aggressive behaviors [22]. Nowadays, several studies provide strong evidence for the existence of a cellular subpopulation with stem-like traits also in NSCLC [20, 22, 23], where the presence of these cells predicts a worse prognosis for patients [24]. Currently, the development of new treatment strategies that target CSCs, as well as the identification of new druggable pathways, are the main goals of anti-cancer therapy [25–34]. Commercially available established cancer cell lines cannot account for the genetic diversity among patients or for the heterogeneity of tumor cells. Novel approaches directed at eradicating CSCs could be greatly strengthened by the use of patient-derived CSCs as cellular model for the discovery of compounds that can selectively target these cells [35]. In this context, we investigated the antitumor efficacy of CPTH6 using *in vitro* and *in vivo* models of spheroid patient-derived lung CSCs (LCSCs).

## RESULTS

### CPTH6 inhibits *in vitro* cell viability of human NSCLC cell lines

**Figure 1:**
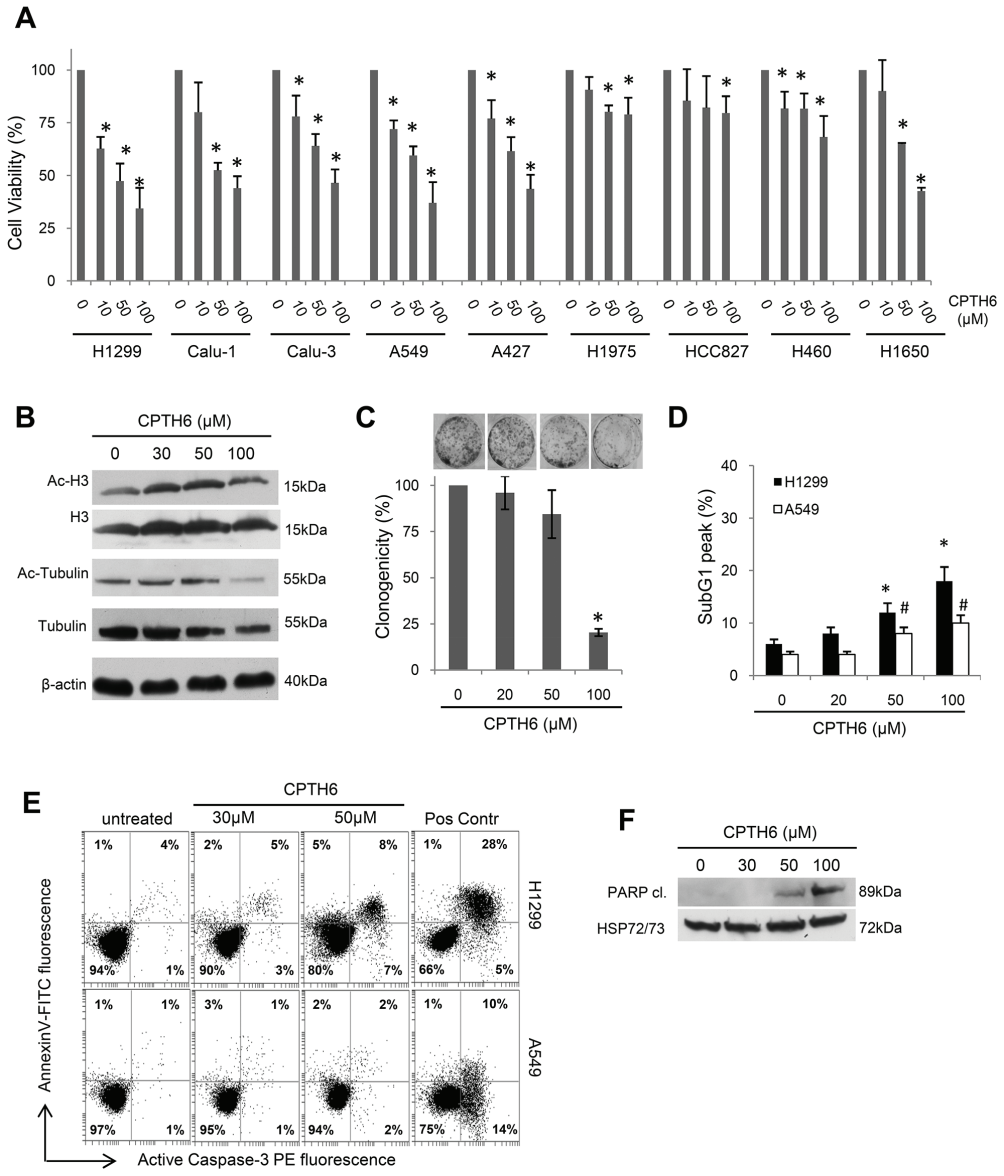
CPTH6 inhibits *in vitro* cell viability of human NSCLC cell lines **A.** Analysis of cell viability by MTT assay in the indicated established NSCLC cell lines exposed to CPTH6 concentrations ranging from 10 to 100μM for 72h. **B.** Western Blot analysis of α-tubulin, histone H3, acetylated α-tubulin (Ac-Tubulin) and histone H3 (Ac-H3) levels in H1299 cells treated for 24h with CPTH6 at the indicated concentrations. β-actin is shown as loading and transferring control. **C.** Representative images and quantification of colony assay performed on H1299 cells untreated or treated for 24h with CPTH6 at the indicated concentrations. Percentage of clonogenicity relative of treated versus untreated cells is reported. **D.** Flow cytometric quantification of sub-G1 DNA peak by propidium iodide staining in H1299 and A549 cells untreated or treated with CPTH6 for 72h at the indicated concentrations. **E.** Flow cytometric analysis of apoptotic cells by AnnexinV/caspase-3 staining in H1299 and A549 cells untreated or treated for 72h with CPTH6 at the indicated concentrations. Treatment with cisplatin (20μM) for 24h represents positive control (Pos Contr). **F.** Western Blot analysis of PARP cleavage in H1299 cells treated for 72h with CPTH6 at the indicated concentrations. HSP72/73 is shown as loading and transferring control. (A) The results are reported as “viability of drug-treated cells/viability of untreated cells” × 100 and represent the average ± SD of three independent experiments. (B, F) Western Blots representative of two independent experiments with similar results are shown. (A, C, D) The results represent the average ± SD of three independent experiments. p-values were calculated between untreated and treated cells. ^#^,*p<0.01.

To evaluate the specific functional significance of HAT inhibition in human NSCLC, we explored cell proliferation of nine commercially available established NSCLC cell lines exposed to increasing concentrations of CPTH6, a novel Gcn5 and pCAF HAT inhibitor [12]. Cell lines were differentially sensitive to CPTH6 treatment with IC_50_ values at 72h ranging from 65 to 205μM (73μM for A549, 65μM for H1299, 77μM for Calu-1, 81μM for A427, 85μM for Calu-3, 205μM for HCC827, 147μM for H460, 198μM for H1975, 83μM for H1650) (Figure 1A, Supplementary Figure S1A). Consistent with the HAT inhibitory activity of CPTH6 [12], decreased acetylation of both histone H3 and α-tubulin was observed in H1299 cells, among the most sensitive cell lines, by Western blot analysis after 24h treatment with CPTH6 (Figure 1B). In order to investigate whether CPTH6 inhibition of cell viability was associated with cell death in NSCLC cells, H1299 cells were treated with CPTH6 for 24h at concentrations ranging from 20 to 100μM, and cell survival was assessed. As reported in Figure 1C, after CPTH6 exposure the colony formation capacity was impaired when compared to untreated cells in a dose-dependent fashion. In particular, CPTH6 at 100μM induced a significant decrease of about 80% cell colony formation compared with untreated controls. Of note, at the higher concentrations reduction of cell viability was accompanied by the presence of Sub-G1 peak, annexin-V binding, pro-caspase 3 activation and cleavage of PARP, all parameters indicative of apoptosis (Figure 1D, 1E, 1F, Supplementary Figure S1B). Similarly, CPTH6 induced apoptosis in less than 10% of A549 cells (Figure 1D, 1E), even when they were exposed to 5 days treatment with CPTH6 (data not shown).

### CPTH6 inhibits *in vitro* cell viability of patient-derived lung cancer stem-like cells (LCSCs)

**Figure 2:**
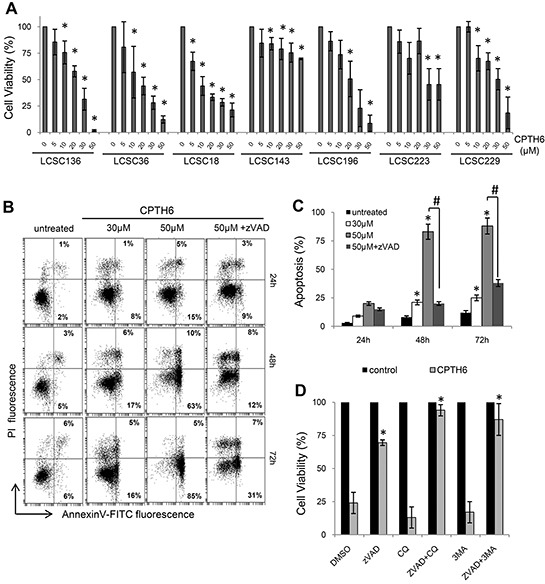
CPTH6 inhibits *in vitro* cell viability and self-renewal of patient-derived lung cancer stem-like cells (LCSCs) **A.** Analysis of cell viability by CellTiter-Glo assay in the indicated patient-derived LCSC lines exposed to increasing concentrations of CPTH6 for 72h. **B, C.** Flow cytometric analysis of apoptotic cells by AnnexinV/PI staining in LCSC136 cells untreated or treated with CPTH6 at the indicated concentrations and exposure times in presence or absence of the pan-caspases inhibitor zVAD-fmk (zVAD, 50μM). (B) The percentage of AnnexinV+/PI− (early apoptotic cells, lower right) and AnnexinV+/PI+ (late apoptotic cells, upper right), cells is shown. A representative experiment out of three with similar results is shown. (C) The results represent the average ± SD of three independent experiments. p-values were calculated between untreated and treated cells. ^#^,*p<0.01. **D.** Analysis of cell viability by CellTiter-Glo assay in patient-derived LCSC136 line untreated or treated with CPTH6 (30μM, 72h) in presence or absence of zVAD (50μM), Chloroquine (CQ, 25μM) and 3-methyladenine (3-MA, 0.5mM). (A, D) The results are reported as “viability of drug-treated cells/viability of untreated cells” × 100. (A, D) The results represent the average ± SD of three independent experiments. p-values were calculated between untreated and treated cells. *p<0.01.

Patient-derived cancer cells, isolated from NSCLC surgical specimens, are undifferentiated and highly clonogenic cells that are resistant to conventional chemotherapy [21]. LCSCs, cultured in serum-free medium containing EGF and basic-FGF in low adherent plate, grow as multicellular spheroids with properties of CSCs, as determined by highly tumorigenicity and expression of stem cell markers (Supplementary Table S1). These spheroid LCSCs represent a suitable cellular model to search new therapeutic options for lung cancer and to account for the genetic diversity among patients, or for the heterogeneity of tumor cells. To this aim, spheroid LCSC lines were exposed to increasing concentrations of CPTH6 for 72 h. We found that CPTH6 had a stronger and significantly growth-inhibitory effect in these patient-derived spheroid cell lines than in established NSCLC lines (Figure 2A, Supplementary Figure S1C), with IC_50_ values ranging from 12 to 67μM (21μM for LCSC136, 23μM for LCSC36, 12μM for LCSC18, 36μM for LCSC196, 25μM for LCSC223, 29μM for LCSC229, 67μM for LCSC143). Of note, a dose- and time-dependent reduction of cell viability associated with an increased percentage of cells in Sub-G1 phase was well evident in the LCSC136 representative line after CPTH6 treatment (Supplementary Figure S2A, S2B).

To better explore the mechanism of CPTH6 cytotoxicity in LCSCs, we quantified the amount of apoptotic cells after treatment. As shown in Figure 2B, 2C, a dose- and time-dependent induction of apoptotic cell death by CPTH6 treatment was well evident in LCSC136 line. In particular, the percentage of annexinV+ cells dose-dependently increased up to about 30% and 80% in LCSC136 cells treated for 72h with 30 and 50μM, respectively. Similar results were obtained with LCSC36 line (data not shown). In agreement with these data, PARP cleavage and phosphorylation of H2AX (γH2AX) were already observed after exposure of LCSC136 line to 30μM CPTH6 for 72h, confirming the occurrence of apoptosis and DNA damage, respectively, in this line (Supplementary Figure S2C).

The addition of the pan-caspase inhibitor zVAD-fmk to LCSC136 line significantly diminished CPTH6-induced annexinV+ cells compared to cells that were treated with CPTH6 in the absence of zVAD-fmk (Figure 2B, 2C). Caspases inhibition, at least in part, prevented the cytotoxic effect of CPTH6 in LCSC136 cells, partially restoring the viability of cells exposed to drug (Figure 2D).

This result suggests that additional cell death mechanisms might be induced by CPTH6. As we previously reported the ability of CPTH6 to modulate autophagy in established tumor cell lines of different origin [11], we determined whether autophagy may play a role in CPTH6 cytotoxicity observed in LCSCs. To this aim, LCSCs were treated for 72h with CPTH6 in the presence of autophagy and apoptosis inhibitors, or with a combination of the two (Figure 2D). Notably, treatment of cells with an early (3-methyladenine) or late (chloroquine) stage autophagy inhibitor did not significantly affect CPTH6-cytotoxic effect. On the other hand, the simultaneous presence of autophagy and apoptosis inhibitors was able to block CPTH6-induced death, indicating that cell death occurs through mechanisms that involves both pathways (Figure 2D).

Next, we were interested to find out whether there was any synergistic or additive effect of CPTH6 when combined with cisplatin or pemetrexed, the current first line chemotherapy agents for NSCLC patients [1]. As shown in Supplementary Figure S2D, combined treatment (ratio 10:1) of low dose of CPTH6 and cisplatin or pemetrexed induced in LCSC136 a decreased viability compared with single treatment. The combination index scores were 0.6 and 0.87 for pemetrexed/CPTH6 and cisplatin/CPTH6 respectively, suggesting a synergistically increased cytotoxicity of combined treatment compared with single ones.

### CPTH6 induces the loss of CSCs expression markers in LCSC36 line

**Figure 3:**
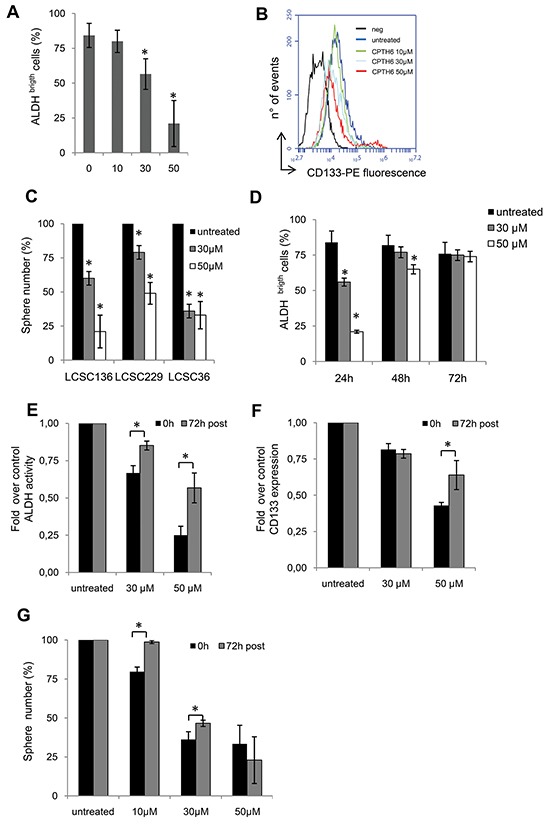
CPTH6 induces the loss of CSCs expression markers in LCSC36 line **A.** Flow cytometric quantification of ALDH activity in LCSC36 cells untreated or treated with CPTH6 for 24h at the indicated concentrations. **B.** Flow cytometric analysis of CD133 in LCSC36 cells untreated (blue) or treated with CPTH6 (10μM green, 30μM grey, 50μM red) for 24h. Black line represents negative control (neg, no addition of primary antibody to the cells). A representative experiment out of three with similar results is shown. **C.** Quantification of tumorsphere formation of the indicated LCSC lines treated for 24h with the indicated concentrations of CPTH6. **D.** Flow cytometric quantification of ALDH activity in LCSC36 cells untreated or treated with CPTH6 at the indicated concentrations and times. **E, F.** Flow cytometric quantification of (E) ALDH activity and (F) CD133 expression in LCSC36 cells treated with the indicated concentrations of CPTH6 for 24h (0h) or after 72h of treatment withdraw (72h post). **G.** Quantification of tumorsphere formation of LCSC36 line treated as reported in (E, F). (C, G) Data shown represent the percentage of spheres normalized to the number of seeded cells. (A, C-G) The results represent the average ± SD of three independent experiments. *p<0.01. (A, C, D) p-values were calculated between untreated and treated cells. (E-G) p-values were calculated between 0h and 72h post.

To evaluate the effects of CPTH6 on CSCs phenotype, we analyzed the change in expression levels of CD133 and ALDH activity in LCSC36 line, characterized by CD133 positivity and high ALDH activity, upon exposure to CPTH6. As shown in Figure 3A, 3B 24h of CPTH6 treatment resulted in downregulation of both stem cell markers in a dose-dependent manner. To test the functional consequences of CPTH6-induced loss of the CSCs expression markers, we used a tumorsphere assay to measure the self-renewal of CSCs [36]. Spheroids LCSCs were treated with CPTH6 for 24h, and the effect of treatment on CSCs pool was determined in a secondary tumorsphere assay (Figure 3C, Supplementary Figure S3D). Intriguingly, treatment resulted in a reduction of self-renewal capacity *in vitro* in a dose-dependent manner in LCSC36 and in other two LCSC lines.

To investigate whether CPTH6 results in a reduction of stemness properties independently of its ability of triggering cell death, we performed a time course experiment to follow change in ALDH activity upon CPTH6 treatment. To this aim, cells were analyzed by gating on forward and side scatters as well as viability to exclude dead cells. As shown in Figure 3D, at 72h CPTH6 treatment was not effective in downregulating stem cell marker in the alive population. Next, we treated spheroid cultures for 24h with CPTH6 and evaluated the change in expression levels of CD133 and ALDH activity (Figure 3E, 3F) and tumor spheres formation (Figure 3G) at the end of treatment or after 72h from CPTH6 wash out. After 72h of treatment withdraw, we found a significantly induction of ALDH-positive cells content and CD133 expression in viable LCSCs compared to the content found in LCSCs directly after the treatment. Similarly, we found that the CPTH6-induced loss of self-renewal capacity was reversed at 72h of treatment withdraw. Surprisingly, no significant difference were observed at highest dose, likely for high content of apoptotic cells (about 77%) in spheroid cultures at this time. These data suggest that LCSCs disappeared from the spheroid cultures by a process of cell death and that CPTH6 effect on CSCs pool is reversible.

### HAT inhibition preferentially affects cell viability of patient-derived LCSC stem counterpart

**Figure 4:**
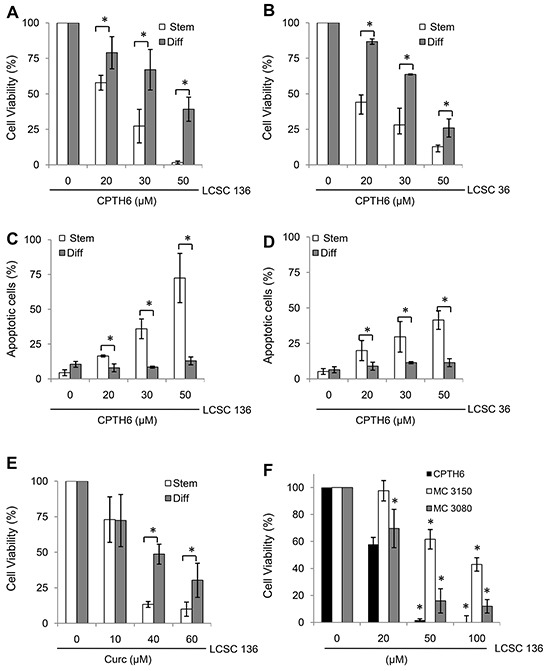
HAT inhibition preferentially affects cell viability of LCSC stem counterpart **A, B.** Analysis of cell viability by CellTiter-Glo assay in patient-derived LCSC136 (A) and LCSC36 (B) spheroids (Stem) and their differentiated progeny (Diff) exposed to increasing concentrations of CPTH6 for 72h. **C, D.** Flow cytometric quantification of apoptotic cells by AnnexinV/PI staining in LCSC136 (C) and LCSC36 (D) spheroid cells (Stem) and their differentiated progeny (Diff) treated for 72h with CPTH6 at the indicated concentrations. **E.** Analysis of cell viability by CellTiter-Glo assay in patient-derived LCSC136 spheroids (Stem) and their differentiated progeny (Diff) exposed to increasing concentrations of pan-HAT inhibitor Curcumin (Curc) for 72h. **F.** Analysis of cell viability by CellTiter-Glo assay in patient-derived LCSC136 spheroids cells exposed to increasing concentrations of CPTH6, and of CPTH6-analogues MC 3080 and MC 3150 for 72h. (A-F) The average ± SD of three independent experiments is shown. p-values were calculated between spheroid and differentiated cells. *p<0.01

We next compared the effect of CPTH6 on the survival of LCSCs grown as undifferentiated multicellular spheroids (Stem) or as differentiated cells in adherent conditions (Diff). Under the latter condition, lung cancer spheres adhered to the plastic and acquired the typical morphologic features of differentiated cells and lost the expression of CD133, confirming the specific expression of this antigen in undifferentiated cells (Supplementary Figure S3A, S3B). As shown in Figure 4, differentiated progeny of both LCSC136 (Figure 4A, 4C) and LCSC36 (Figure 4B, 4D) cells were more resistant to CPTH6 cytotoxicity when compared to their stem counterparts. As expected, 72h of CPTH6 exposure increased the percentage of apoptotic cells in both LCSC136 and LCSC36 cells in a dose-dependent manner when grown as tumor spheroids, whilst less than 10% of cell death was observed in CPTH6-treated differentiated progeny. Of note, accordingly with their stemness features, undifferentiated LCSCs were more resistant to commonly used antineoplastic agents, such as cisplatin (DDP), when compared to their differentiated progeny, which were overall more sensitive to drug-induced death (Supplementary Figure S3C).

To further address the importance of HAT inhibition in LCSC survival, we analyzed the effect of both p300/CBP HAT inhibitors Curcumin [37] and C646 [38] on cell viability of LCSC136 line grown as undifferentiated multicellular spheroids (Stem) or as differentiated in adherent conditions (Diff). Interestingly, both Curcumin (Figure 4E) and C646 (Supplementary Figure S4B) reduced cell viability of LCSC136 cells grown as tumor spheroids, whilst differentiated progeny were more resistant to either Curcumin or C646 induced cell death (Figure 4E, Supplementary Figure S4B). We next analyzed the activity of two CPTH6 analogues, MC 3150 and MC 3080 (Supplementary Figure S4A, 4F), which displayed very poor pCAF, but a p300 HAT enzyme inhibiting activity higher than prototype [8]. As reported in Figure 4F cell viability revealed that both analogues were less potent than CPTH6 in terms of growth inhibition on spheroids LCSC136 line. Of note, both compounds only very slightly affected the viability of differentiated comparted of LCSCs (Supplementary Figure S4B). Finally, the effect of different HAT inhibitors on CSCs was determined in a secondary tumorsphere assay (Supplementary Figure S4C), confirming the importance of HAT enzymes in the self-renewal of CSCs.

### CPTH6 effect on cell viability correlates, not only to the extent to which protein hypoacetylation occurs, but also to the baseline level of acetylated α-tubulin

**Figure 5:**
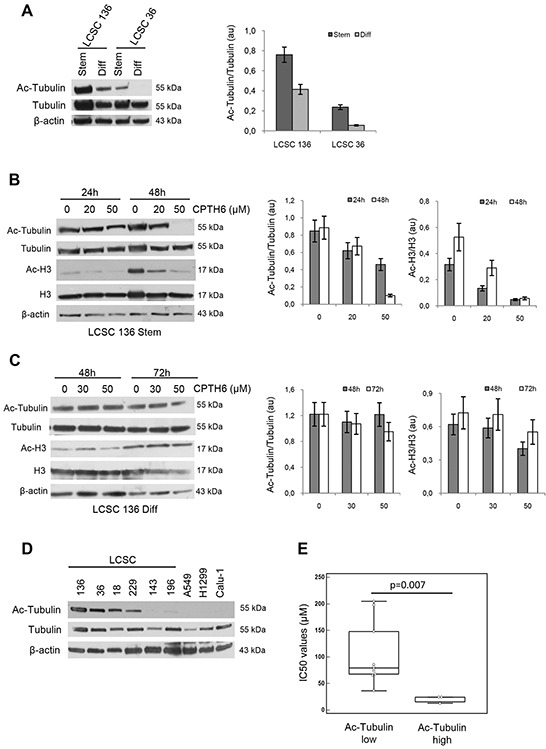
CPTH6 effect correlates, not only to the extent to which protein hypoacetylation occurs, but also to baseline level of acetylated α-tubulin **A.** Western Blot (left panel) and relative densitometric analyses (right panel) of α-tubulin and acetylated α-tubulin (Ac-Tubulin) in LCSC136 and LCSC36 lines grown as spheroid cells (Stem) or in adherent conditions (Diff). **B, C.** Western Blot (left panel) and relative densitometric (right panel) analyses of α-tubulin, histone H3, acetylated α-tubulin (Ac-Tubulin) and histone H3 (Ac-H3) levels in LCSC136 spheroid (B) and LCSC136 differentiated (C) cells treated with CPTH6 at the indicated concentrations and times. Protein levels were quantified by densitometric analyses and ratio between protein of interest and loading control is presented. **D.** Western Blot analysis of α-tubulin and acetylated α-tubulin (Ac-Tubulin) in the indicated established NSCLC and patient-derived LCSC lines. **E.** Correlation between acetylated α-tubulin level (high, low) and CPTH6 sensitivity (IC50 values) by Mann-Whitney test in NSCLC and patient-derived LCSC cells. The y-axis shows the CPTH6 concentration causing a 50% inhibition of cell viability (IC50). p value is 0.007. (A-D) β-actin is shown as loading and transferring control. Western blots representative of two independent experiments with similar results are shown.

Next, we tested whether CPTH6 effect on undifferentiated and differentiated cells may be correlated with baseline expression of acetylated protein. Notably, Western blot analysis showed that both LCSC136 and 36 cells grown as tumor spheroids have higher level of acetylated α-tubulin into K40 residue when compared to their differentiated progeny (Figure 5A). We also observed that CPTH6 treatment induced a significant time- and dose- dependent decrease of acetylated α-tubulin and histone H3 in stem LCSC136 cells. In particular, we observed a decrease of about 45% and 85% of histone H3 and α-tubulin acetylation, respectively upon 24h of treatment with 50μM CPTH6 (Figure 5B). On the contrary, the differentiated progeny was more resistant to reduction of protein acetylation, in fact the higher concentration of CPTH6 50μM induced a reduction of about 2% and 35% in the level of acetylated tubulin and of about 23% and 35% in the level of acetylated histone H3 after 48h and 72h, respectively (Figure 5C). These results suggest that CPTH6 effect on sensitive cells may be predicted by baseline level of acetylated α-tubulin. Hence, in order to investigate whether baseline levels of acetylated α-tubulin could be related to sensitivity to CPTH6, intracellular baseline levels of acetylated α-tubulin were quantified in LCSC (n=6) and NSCLC (n=8) lines. As reported by Western blot analysis, α-tubulin was prominently acetylated in 4 out of 6 LCSC lines which are consistently more sensitive to the growth-inhibitory effects of CPTH6 (Figure 5D, 5E). Such observation raises the interesting possibility that acetylated α-tubulin might represent a novel marker of sensitivity to HAT inhibition.

### CPTH6 reduces the *in vivo* growth of LCSCs-derived tumor xenografts

**Figure 6:**
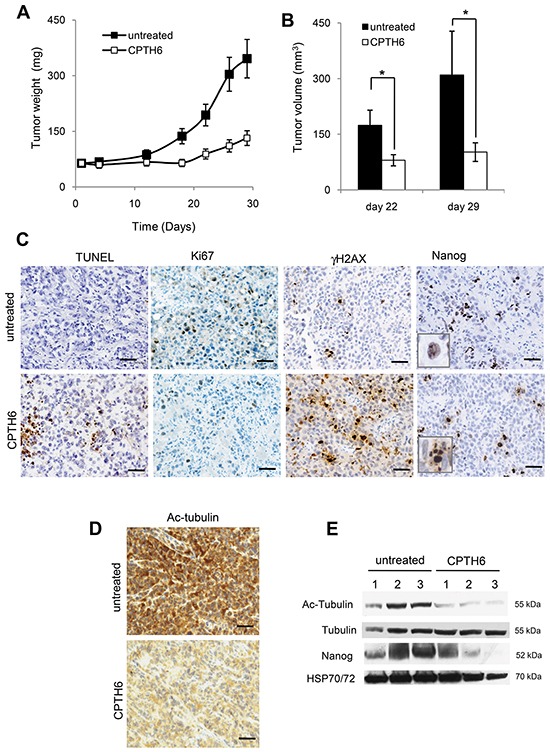
CPTH6 reduces the *in vivo* growth of LCSCs-derived tumor xenografts **A, B.** Analysis of *in vivo* tumor growth in NOD/SCID mice inoculated subcutaneously with 2.5×10^5^ LCSC136 cells and treated i.p. with CPTH6 (50mg/Kg; 5 days every 24h for 3 weeks). **C, D.** Representative images of immunohistochemical detection of (C) apoptosis by TUNEL assay, proliferation by Ki67 staining, DNA damage by γH2AX protein staining and Nanog protein expression (D) and acetylated α-tubulin (Ac-Tubulin) level in LCSC136-derived tumors from untreated or treated mice with CPTH6 as reported in (A). Magnification 200×. Scale bar, 100μm. **E.** Western Blot analysis of α-tubulin and Nanog proteins expression and acetylated α-tubulin (Ac-Tubulin) level in LCSC136-derived tumors lysates from untreated or treated mice with CPTH6 as reported in (A). HSP72/73 is shown as loading and transferring control. Western blots representative of two independent experiments with similar results are shown. (B) The average ± SD of two independent experiments is shown. p-values were calculated between untreated and treated tumors. *p<0.01.

We next evaluated the effect of CPTH6 on *in vivo* tumor growth of patient-derived LCSC136 cell line. We have previously demonstrated the high bioavailability of CPTH6 [12], moreover daily treatment with CPTH6 did not produce any adverse health effects as monitored by diet consumption, body weight loss, toxic death and postural and behavioral changes in mice. To determine the *in vivo* antitumor efficacy of CPTH6, LCSC136 spheroids cells were injected subcutaneously into immunocompromised (NOD/SCID) mice. As showed in Figure 6A, 6B and according to *in vitro* data, CPTH6 treatment strongly reduced tumor growth in LCSC136 xenografts. We next evaluated by immunohistochemical analysis of tumor tissues, the impact of CPTH6 treatment on cell proliferation (Ki67), apoptosis (TUNEL) and DNA damage (γH2AX) (Figure 6C). Decreased proliferation and enhanced apoptosis were observed in treated group, in particular, the proliferation was 21.6% in the untreated group versus 8.8% in the treated one, and apoptotic index increased from 3.65% in the untreated group to 35.5% in the treated one, indicating that CPTH6 had a strong anti-proliferation and apoptosis-inducing effects (p<0.05). LCSC136 xenografts also displayed increased γH2AX labeling, confirming a drug-induced DNA damage response in tumor specimens. We also analyzed the effect of CPTH6 on protein acetylation in tumor specimens recovered after 3 weeks of *in vivo* treatment. Accordingly to our *in vitro* results, we confirmed the ability of CPTH6 to inhibit α-tubulin acetylation also *in vivo* (Figure 6D). Cellular lysates from CPTH6-treated tumors displayed a reduction of α-tubulin acetylation levels when compared to that of untreated tumors, thus confirming IHC results and linking the acetyltransferase inhibition activity to the antitumor activity in xenografts (Figure 6E).

To gain more insights into the ability of CPTH6 to target LCSCs *in vivo*, we decided to perform immunohistochemical and Western Blot analyses for the CSC marker Nanog which is well expressed in this LCSC136 line. We found only a slight difference in the number of Nanog-positive cells in CPTH6-treated xenografts when compared to untreated xenografts. Nevertheless, Nanog-positive cells in CPTH6-treated samples but not in untreated ones, were often associated to the presence of apoptotic bodies, thus suggesting that treatment induces LCSCs death *in vivo*. We subsequently determined whether similar preferential targeting of CSCs occurs also *in vivo*, by analyzing tumor-initiating frequency (TIF) in a limiting dilution assay. To this aim after two weeks of treatment, cells dissociated from either CPTH6- or vehicle-treated tumors were injected into NOD/SCID mice in limiting dilution to evaluate the tumor forming ability (Table 1). Of note, cells from CPTH6-treated tumors displayed a 5-fold reduction of TIF, confirming reduction of CSCs in tumors by CPTH6 treatment.

**Table 1:** *In vivo* limiting diluition assay showing that CPTH6 reduced tumor-initiating capability of LCSC136

Cells (n)	Untreated	CPTH6
250000	9(8)	9(3)
50000	9(3)	9(1)
TIF	1/118000	1/570000
	P=0.00532	

Cells dissociated from LCSC 136 tumors were re-implanted in limiting dilutions into flank of NOD/SCID immunodeficient mice. The number of mice that grew tumors at week 8 out of total of 9 mice per group (in parenthesis) is tabulated. Tumor Initiating formation (TIF) was calculated using the ELDA software.

## DISCUSSION

Several epigenetic inhibitors have been developed and shown to induce differentiation, growth arrest, or apoptosis in tumor cells [7–10, 39–41]. Among them, we previously characterized the thiazole derivative CPTH6, as a novel HAT inhibitor that activates the apoptotic program and modulates the autophagic flux in human tumor cell lines [11, 12].

Targeting CSCs to suppress tumor growth is a major focus in current cancer research [26, 28–31, 33, 35, 42–45]. To investigate the functional significance of HAT inhibition in NSCLC and in targeting CSCs, in this study we used a panel of well-characterized established NSCLC and spheroid patient-derived LCSC lines. The latters display several stem cell-associated properties including self-renewal capability, higher clonogenic potential, resistance to chemotherapy and the expression of diverse CSCs markers, such as CD133, ALDH1 (detected as ALDH activity), Nanog and CD44. Noteworthy, none of analyzed markers showed a high and consistent expression in all the spheroids patient-derived LCSC lines, that were used in this study, thus confirming the heterogeneity in lung CSCs marker expression [46–48]. Our results show that CPTH6 preferentially inhibits cell viability and activates the apoptotic program of spheroid LCSCs with properties of CSCs. Interestingly, CPTH6 treatment is more efficient in LCSCs than in NSCLC commercially available cell lines. Unlike LCSC models, in which CPTH6 treatment induces apoptosis even at low doses, in established NSCLC lines CPTH6 triggers a low percentage of apoptotic cells only when high concentrations and time of exposure are used.

CPTH6 induces in spheroid LCSCs a form of cell death that resembles canonical caspases-dependent apoptosis associated with DNA damage as documented by appearance of Sub-G1 peak, annexin-V binding, PARP cleavage, and increased of H2AX phosphorylation. Conceivably, the effect of CPTH6 on DNA damage may be due to its ability to block not only H3 acetylation but also other histone acetylation marks, such as K16 acetylation of H4 histone, that promotes double-strand breaks repair [49] or to influence non-histone acetylated proteins that are involved in DNA damage and repair such as p53, ATM, Ku70 and Exo1 [50]. Noteworthy, single-agent antitumor activity was observed in spheroid LCSC-derived models treated with CPTH6, without signs of toxicity as evidenced by body weight measurement, diet consumption, toxic death and postural and behavioral changes of mice. CPTH6 treatment reduced the growth of LCSC-derived tumors and significantly increased the amount of apoptotic and DNA damaged cells present in the xenografts. By contrast, CPTH6 treatment had only a moderate not significant inhibitory effect and did not induce apoptosis in H1299 xenografts (data not shown). Notably, the preferential targeting of lung population with CSC properties by CPTH6 is also underscored by the evidence that its anti-tumor effect is accompanied *in vivo* by a decrease in stem cell markers, as Nanog. Moreover, Nanog-positive cells in CPTH6-treated samples but not in untreated one, were often associated to the presence of apoptotic bodies, thus suggesting the treatment induces selectively LCSCs death. CPTH6 also preferentially reduced the proportion of cells with self-renewal potential as measured by secondary tumorsphere assays and by tumor-initiating capability following limiting dilution re-implantation of cells into secondary immunodeficient mice. By using different HAT inhibitors, we also provide clear evidence that inhibition of HAT confers a strong preferential inhibitory effect on cell viability of undifferentiated LCSC lines when compared to their differentiated progeny. Of note, two compounds closely related to CPTH6 which displayed higher p300 and very poor pCAF inhibiting activity [8], and the specific p300 inhibitor C646, were less/comparably potent than CPTH6 in terms of growth inhibition on spheroids but very slightly affected the viability of differentiated LCSC.

Our results are also in agreement with papers reporting that epigenetic modulation, using chromatin modifiers, appears as an encouraging means to control CSCs fate [51, 52]. In this context, a dual role of histone acetylation in embryonic stem cell pluripotency maintenance and differentiation [24], as well as the ability of several epigenetic compounds to modulate stem cells and tumor cell differentiation, has been shown [53, 54]. CPTH6 effect on cell viability seems to be correlated not only to the extent to which protein hypoacetylation occurs but also to baseline level of acetylated α-tubulin, which was particularly prominent in sensitive LCSCs. LCSC lines are more sensitive than established NSCLC cells not only in terms of loss of cell viability, but also in the decreasing of proteins acetylation. In fact, a different response in decreasing of their acetylation level of H3 histone and α-tubulin after CPTH6 treatment was observed in undifferentiated LCSC lines when compared to their differentiated progeny. Of note, differentiated progeny of LCSC lines have lower level of expression of acetylated α-tubulin when compared to relative LCSCs grown as multicellular spheroids.

Acetylated tubulin is implicated in a broad range of cell functions including intracellular endoplasmic reticulum (ER) localization and ER-mitochondria interactions [55] as well as the regulation of microtubule dynamics [56]. Recently, high level of acetylated tubulin expression have been found correlated with a higher tumor grade in squamous cell carcinoma of the head and neck [57], and its expression has been suggested as a prognostic marker in epithelial malignancies and as a marker for sensitivity to chemotherapy [58]. A relationship between high level of α-tubulin acetylation and metastatic behaviour of basal-like breast cancers, it has been also recently reported [59]. Hence, level of α-tubulin acetylation may represent a reliable biomarker for predicting response to HATi treatment to identify patients likely to benefit from these drugs. Strikingly, the ability of CPTH6 to inhibit tubulin acetylation was confirmed also *in vivo*. Even if further studies should be performed to fully address the possibility that the effects on tubulin acetylation could be responsible for the apoptosis and tumor growth inhibition, emerging data support a role for tubulin in the execution of cell death in response to stress. In particular, tubulin has been demonstrated to interact with regulators of mitochondrial membrane permeability and apoptosis [60]. Tubulin acetylation occurs on lysine 40 (K40) by the α-tubulin acetyltransferase 1 (ATAT1, [61]) an acetyltransferase belonging to the Gcn5 HAT family. Our results showing that α-tubulin levels correlate with drug sensitivity enforce the evidence that ATAT1 could be another direct target of CPTH6 and that level of acetylated tubulin may be a predictive biomarker of response to this class of HAT inhibitors.

Indeed, we cannot exclude that tubulin acetylation status may be a new marker for CSCs. Additional studies using a larger number of patient-derived cells with stem cells features are necessary to determine the significance of α-tubulin acetylation as a CSCs marker.

Overall, we demonstrated CPTH6 antitumor activity in both stem and non-stem cell populations, thus the proposed approach may represent a potentially successful therapeutic strategy from both a classical hierarchical static model of CSCs point of view and a dynamic stemness perspective.

## MATERIALS AND METHODS

### Cell culture and reagents

Human commercially available established NSCLC lines (H1299, H460, A549, H1650, Calu-1, Calu-3, A427, H1975, HCC827) were cultured in 10% inactivated fetal bovine serum (HyClone, Thermoscientific, South Logan, UT) in RPMI medium (EUROCLONE, Milan, IT). LCSC18, LCSC36, LCSC136, LCSC196, LCSC223, LCSC229, LCSC143 lines were isolated from lung patients, and cultured as spheroid cultures in CSCs medium [21, 62]. LCSCs differentiation was obtained by growing cells in adherent conditions for 24h in DMEM 10% plus fetal bovine serum (HyClone, ThermoScientific) and for 3 additional days in Bronchial Epithelial Cell Growth Medium (Cambrex, East Rutherford, NJ, USA). 3-Methylcyclopentylidene-[4-(4′-chlorophenyl)thiazol-2-yl]hydrazone (CPTH6) was dissolved in dimethyl sulfoxide (DMSO, Sigma-Aldrich, St. Louis, MO, USA) and diluted to the final concentrations in complete medium. For all the experiments cells were treated with 1% DMSO, as control. After 24h from seeding, exponentially growing cells were treated with CPTH6 at concentrations ranging from 1 to 100μM for 24-120h. zVAD-fmk (zVAD, 50μM, Sigma-Aldrich), 3-methyladenine (3-MA, 0.5mM, Sigma-Aldrich) and chloroquine (CQ, 25μM, Sigma-Aldrich) were dissolved in DMSO.

### Cell viability, clonogenic and tumorsphere formation assays

The inhibitory effect of CPTH6 on i) NSCLC cell growth by measuring 3-[4,5-dimethylthiazol-2-yl]-2,5-diphenyltetrazolium bromide inner salt (MTT, Sigma-Aldrich) dye absorbance of cells, and ii) spheroids LCSCs cell growth by quantitation of the ATP present in metabolically active cells using CellTiter-Glo® Luminescent (Promega, Fitchburg, Wisconsin, USA). Manifacturer's protocol was followed. To evaluate the cell colony-forming ability, cell suspensions from different samples were seeded into 60-mm Petri dishes for 10 days. Colonies were stained with 2% methylene blue in 95% ethanol and counted (1 colony>50 cells). The surviving fractions were calculated as the ratio of absolute survival of the treated sample/survival of untreated control sample. For combination treatment, cells were treated with each drug, either alone or in combination, as follows: (a) CPTH6 (b) Pemetrexed or cisplatin (c) Pemetrexed or cisplatin followed by CPTH6. Data were analyzed by the median-effect method (CalcuSyn software, Biosoft) to determine the combination index (CI) [62]. For tumorsphere assay, spheroid cultures were recovered after treatment, counted and seeded in 24-well ultra low-attachment surface plates at a density of 5×10^2^ cells/well and cultured as previously described. After 10 days, spheres were photographated and counted. Data shown represent the percentage of spheres normalized to the number of seeded cells.

### Flow cytometric analysis

Cell cycle distribution by PI staining and apoptosis by AnnexinV-FITC (BD biosciences, San Diego, CA, USA)/PI staining were performed as previously described [62]. For analysis of CD133+ subpopulation, the cells were washed with PBS, and then incubated with antibody against CD133/1 conjugated with phycoerythrin (PE; MiltenyiBiotec, BergischGladbach, Germany) on ice in the dark for 20 min. After the cells had been washed with PBS, the CD133^+^ and CD133^−^ populations were analyzed by FACS. Active caspase-3 Apoptosis Kit (BD biosciences) was used to detect the heterodimer of 17 and 12 kDa subunits, which is derived from the pro-enzyme. For annexinV and caspase-3 double staining, cells were washed with 1× binding buffer and then incubated with FITC annexinV (BD biosciences) for 15 min at room temperature in the dark. After annexinV staining, caspase-3-PE staining was performed on the same cells following manufacturer instructions. ALDH activity was evaluated by ALDEFLUOR kit (ALDH, STEMCELL Tecnologies, Vancouver, BC, Canada) following manufacturer instructions. All flow cytometric analyses were performed by using BD Accuri™ C6 flow cytometer (BD biosciences).

### Western blot analysis

Western blot analyses of total protein extracts were performed as previously described [12]. Immunodetection was performed using antibodies directed to: H3 histone (abcam), H3 acetylated histone (Millipore, Billerica, MA, USA), α-tubulin (DM1A) (Santa Cruz Biotechnology, Santa Cruz, CA, USA), acetyl-α-tubulin (K40) (Sigma-Aldrich), γH2AX (Ser139) (Millipore), PARP cleaved (Millipore), PARP (Santa Cruz Biotechnology) β-actin (Sigma-Aldrich), HSP72/73 (Calbiochem, San Diego, CA, USA), anti-mouse or anti-rabbit immunoglobulin G (IgG)-horseradish peroxidase conjugated antibodies (Cell Signaling; Amersham Biosciences, Freiburg, Germany). Antibody binding was visualized by enhanced chemiluminescence method (Amersham Biosciences) according to manufacturer's specification and recorded on autoradiographic film (Amersham Biosciences). Densitometric evaluation was performed using Image J software and normalized with relative controls depending on the Analysis.

### *In vivo* experiments

All procedures involving animals and their care were authorized and certified by the decree n. 67/97A of the Italian Minister of Health and protocol 2560/97 of the Rome Health Service Unit (ASL – RMB)

For *in vivo* experiments, CPTH6 was dissolved in Carboxymethyl cellulose (Sigma, C9481). To evaluate the effect of CPTH6 on tumor growth, 2.5×10^5^ LCSC136 cells were resuspended in 200 μl of matrigel (2.5mg/ml, BD Biosciences, 354234) and injected subcutaneously into 6-8 week-old female immunocompromised (NOD/SCID) mice (10 for each group). For H1299 xenografts, 5×10^6^ cells were injected intramuscularly into 6-8 week-old female immunodeficient athymic nude (nu/nu) mice (10 for each group). Intraperitoneal treatment with CPTH6 (50 or 100mg/Kg) every 24h started when tumors were palpable (about 20 and 80 days after cell injection for H1299 and for LCSC136 respectively), and stopped after three weeks. Mice survival was calculated by euthanizing the animals when the tumors reached 2.0 gr. The experiments were repeated twice. Immediately after sacrifice the formed tumors were removed and cut in two. One half was frozen in liquid nitrogen and stored at −80C for Western blot analysis, and one half was fixed in 4% buffered formalin and routinely paraffin embedded for Immunohistochemistry analysis.

For the limiting dilution assay, cells dissociated from xenograft tumors were admixed with Matrigel and subcutaneously injected in limiting dilutions into NOD/SCID mice to evaluate the tumor forming. Tumor-initiating frequency (TIF) was calculated using the ELDA Software [63].

### Immunohistochemistry (IHC) and *In vivo* Terminal DeoxynucleotidylTransferase-Mediated Nick End Labeling (TUNEL) assay

Phosphorylated form of H2AX, α-tubulin acetylation, Ki-67 and Nanog expression were evaluated, by IHC on paraffin embedded sections cut from tumor xenografts, using the following antibodies: γH2AX(Ser139, Millipore), Ki-67 (MIB-1, Dako, Milan, Italy), Nanog (Cell Signaling), acetyl-α-tubulin (K40) (Sigma-Aldrich). For each tumor, three different 5μm paraffin sections were analyzed and examined by light microscopy. Sections were scanned at 200× magnification. H2AX and Ki-67 nuclear immunoreaction of tumor cells was counted in four high-power fields (400× magnification) per section. For each tumor, counts were averaged to determine the number of positive cells. Immunoreactions were revealed by a streptavidin-biotin enhanced immunoperoxidase technique in an automated autostainer (Bond^TM^ Max, Leica BioSystem). The immunohistochemical detection of apoptosis was performed by TUNEL assay using a commercial kit (In Situ Cell Death Detection Kit, POD, Roche). The assay was performed according to the manufacturer's instructions. For each tumor, three different 5μm frozen sections were analyzed and examined by light microscopy. Sections were scanned at 200× magnification. Apoptosis of tumor cells was counted in four high-power fields (400× magnification) per section. Evaluation of the IHC results was performed independently and in blinded manner by two investigators.

### Statistics

Experiments were replicated three times, unless otherwise indicated, and the data were expressed as average ± standard deviation (SD). Differences between groups were analyzed with a two-sided paired or unpaired *t* test and were considered to be statistically significant for p<0.05. Mann-Whitney test were used for correlation studies.

## SUPPLEMENTARY FIGURES AND TABLE


