# Supplementary material for: Histone acetyltransferase inhibitor CPTH6 preferentially targets lung cancer stem-like cells
Source: Oncotarget. 2016 Feb 8;7(10):11332–48. doi: 10.18632/oncotarget.7238 (PMC4905477; doi:10.18632/oncotarget.7238)
Supplement: Supplementary file 1 [file oncotarget-07-11332-s001.pdf]

SUPPLEMENTARY FIGURES AND TABLE

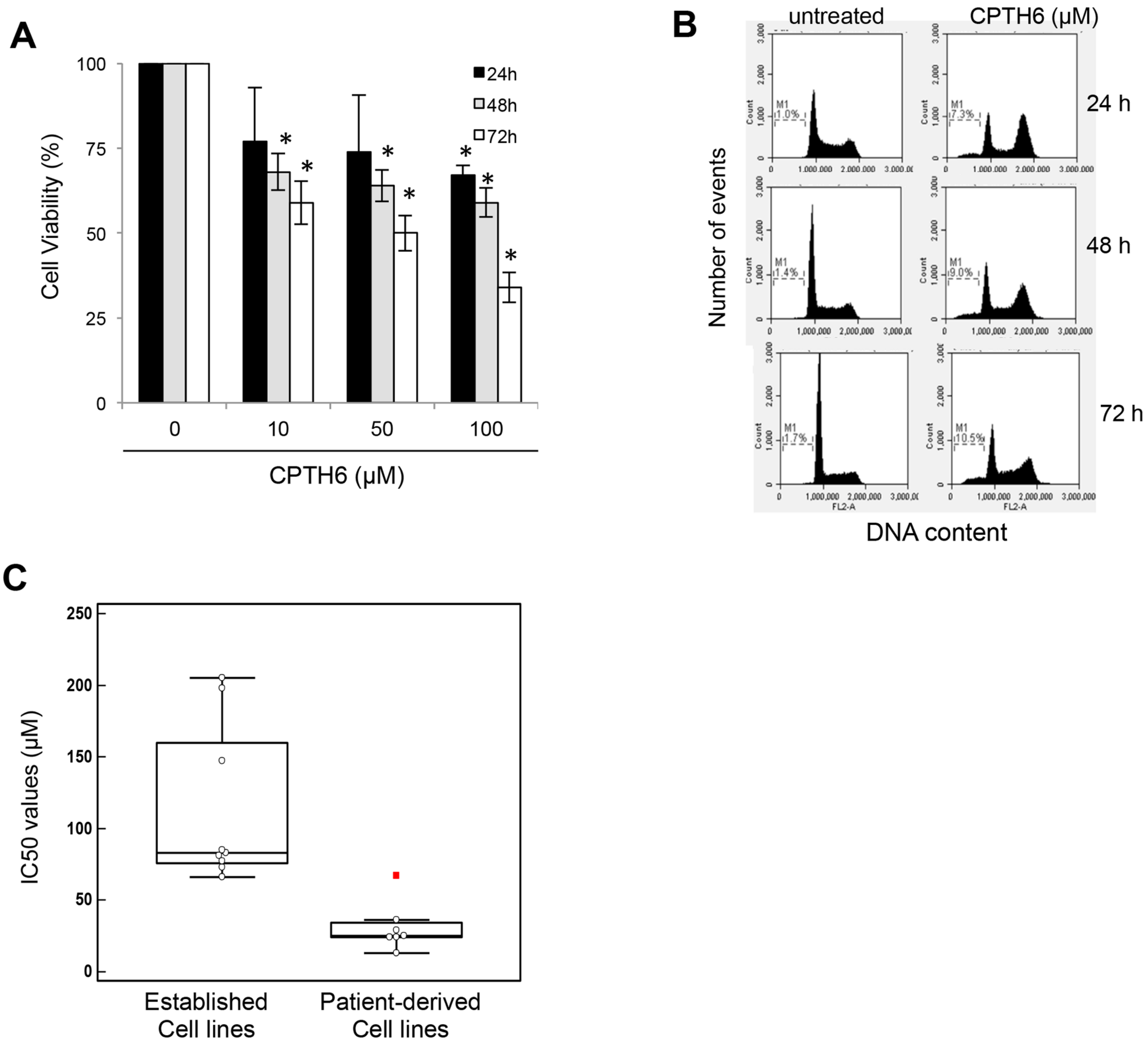

Supplementary Figure S1: Effect of CPTH6 on H1299 cell viability and cell cycle progression A. Analysis of cell viability by MTT assay in H1299 cells exposed to increasing concentrations of CPTH6 for time ranging from 24h to 72h. The results are reported as "viability of drug-treated cells/viability of untreated cells" × 100 and represent the mean ± SD of three independent experiments.*p<0.01. **B.** Flow cytometric analysis of cell cycle distribution by propidium iodide staining in H1299 cells untreated or treated with CPTH6 (50μM) for times ranging from 24h to 72h. The percentage of sub-G1 DNA peak (M1) is shown. **C.** CPTH6 sensitivity in all cell lines analyzed are compared by cell type using Mann-Whitney test. The y-axis shows the CPTH6 concentration causing a 50% inhibition of cell viability (IC50). p-value is 0.0003.

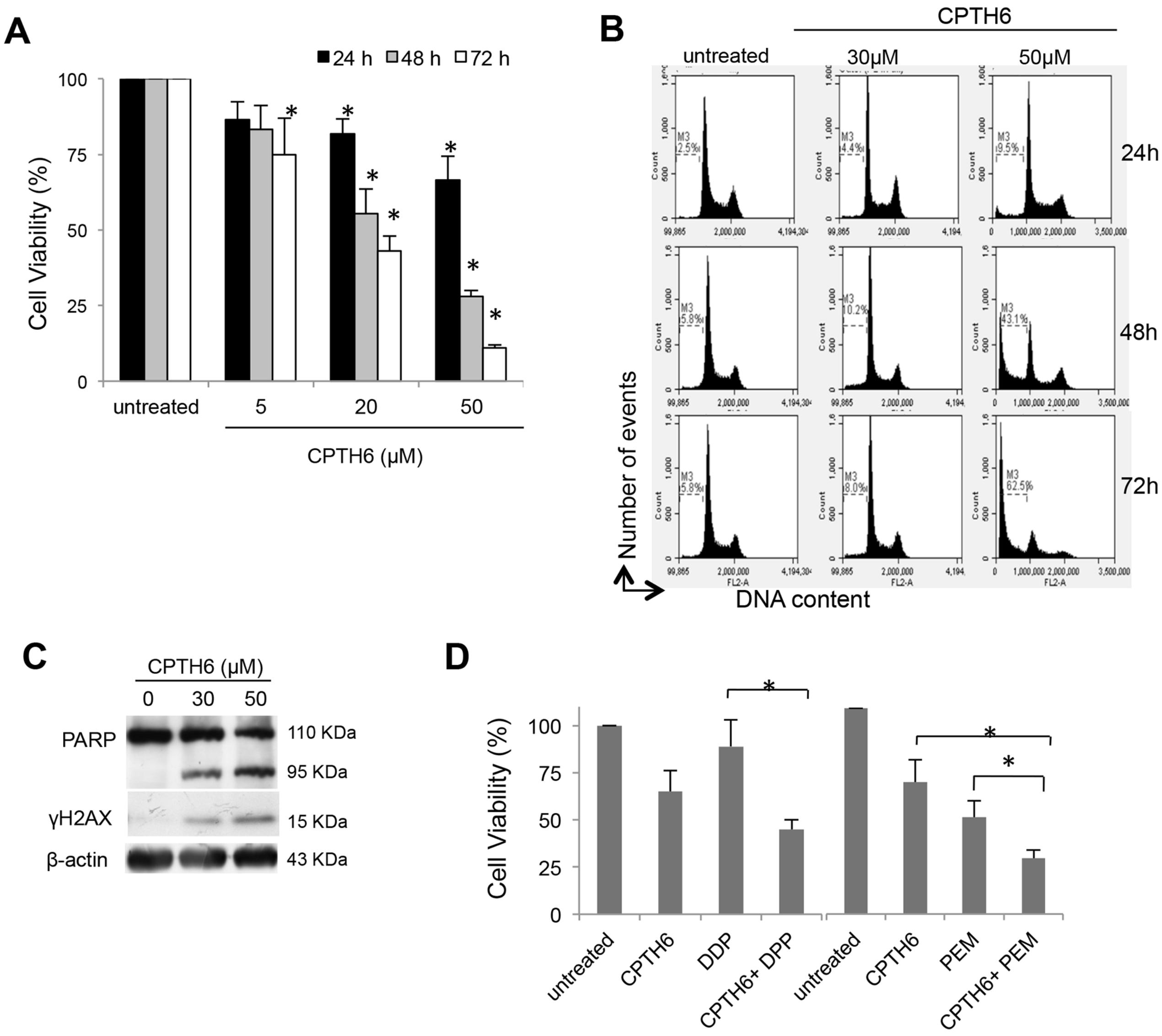

Supplementary Figure S2: Effect of CPTH6 on LCSC136 viability alone or in combination with chemotherapy. **A.** Analysis of cell viability by CellTiter-Glo assay in LCSC136 line exposed to increasing concentrations of CPTH6 for time ranging from 24h to 72h. **B.** Flow cytometric analysis of cell cycle distribution by propidium iodide staining in LCSC136 cells untreated or treated with indicated concentrations of CPTH6 for times ranging from 24h to 72h. The percentage of sub-G1 DNA peak (M3) is shown. **C.** Western Blot analysis of PARP cleavage and phosphorylated γH2AX protein (ser139) expression in LCSC136 cells treated for 72h with indicated concentrations of CPTH6. β-actin is shown as loading and transferring control. Western Blot representative of two independent experiments with similar results are shown. **D.** Analysis of cell viability by CellTiter-Glo assay in LCSC136 line exposed to CPTH6 (25μM) alone or in combination with cisplatin (DDP, 2.5μM) or pemetrexed (Pem, 2.5μM) for 72h. (A, D) The results are reported as "viability of drug-treated cells/viability of untreated cells" × 100 and represent the mean ± SD of three independent experiments. *$p<0.01$.

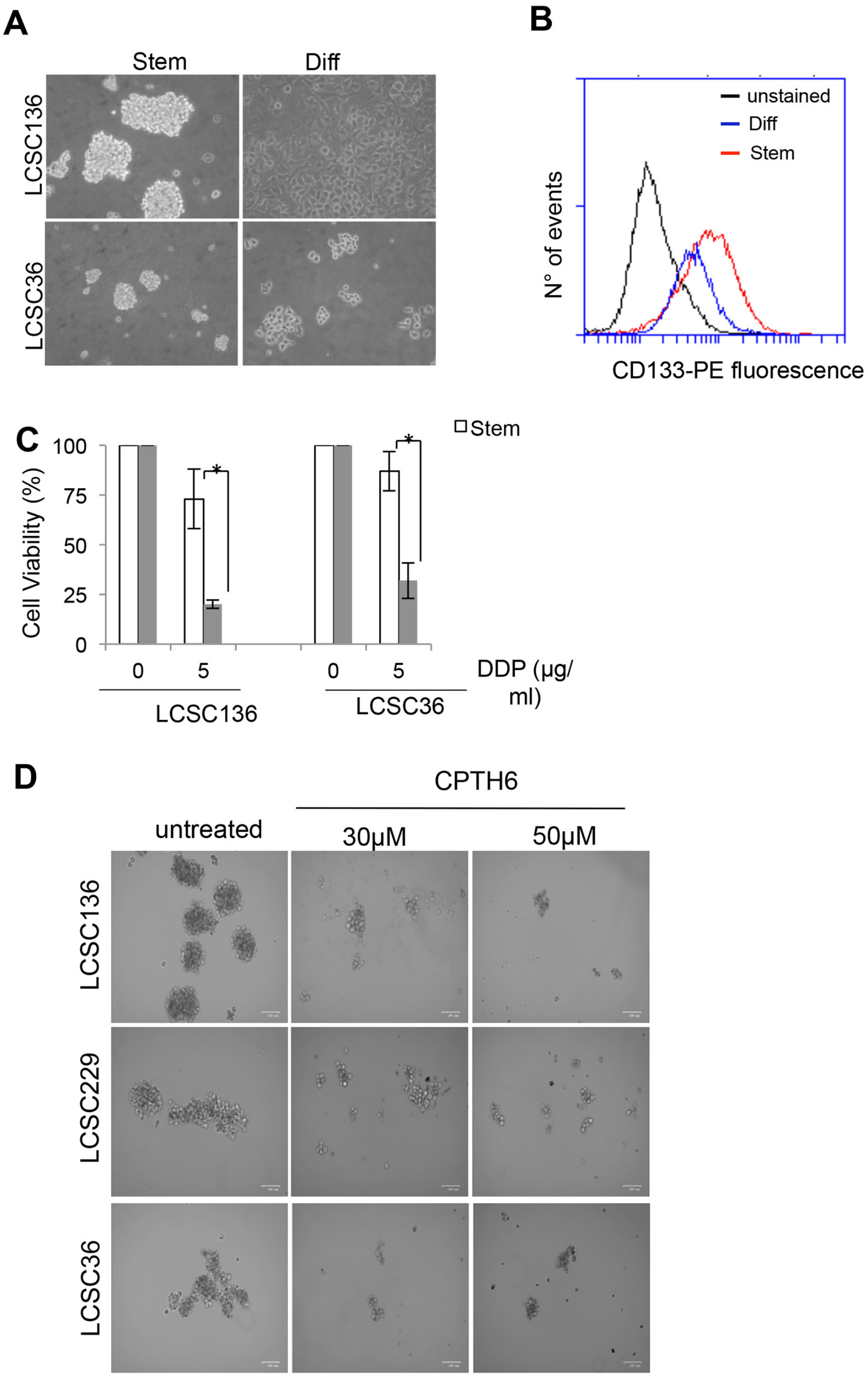

Supplementary Figure S3: Characterization of LCSCs and their resistance to conventional chemotherapeutic drugs. **A.** Light microscopy images of LCSC136 and LCSC36 spheroid cells (Stem) and their differentiated progeny (Diff). Scale bar, 50µm. **B.** Flow cytometric analysis of CD133 in LCSC36 cells (Stem) and their differentiated progeny (Diff). Black line represents negative control (neg, no addition of primary antibody to the cells). **C.** Analysis of cell viability by CellTiter-Glo assay in LCSC136 and LCSC36 spheroid cells (Stem) and their differentiated progeny (Diff) untreated or treated with Cisplatin (DDP, 5µg/ml) for 72h. The results are reported as "viability of drug-treated cells/viability of untreated cells" × 100 and represent the average ± SD of three independent experiments. p-values were calculated between spheroid and differentiated cells. *$p<0.01$. **D.** Analysis of self renewal by tumorsphere formation of LCSC lines. Phase contrast images of tumorsphere formation of the indicated LCSC lines treated for 24h with CPTH6 at the indicated concentrations. Scale bar, 100µm.

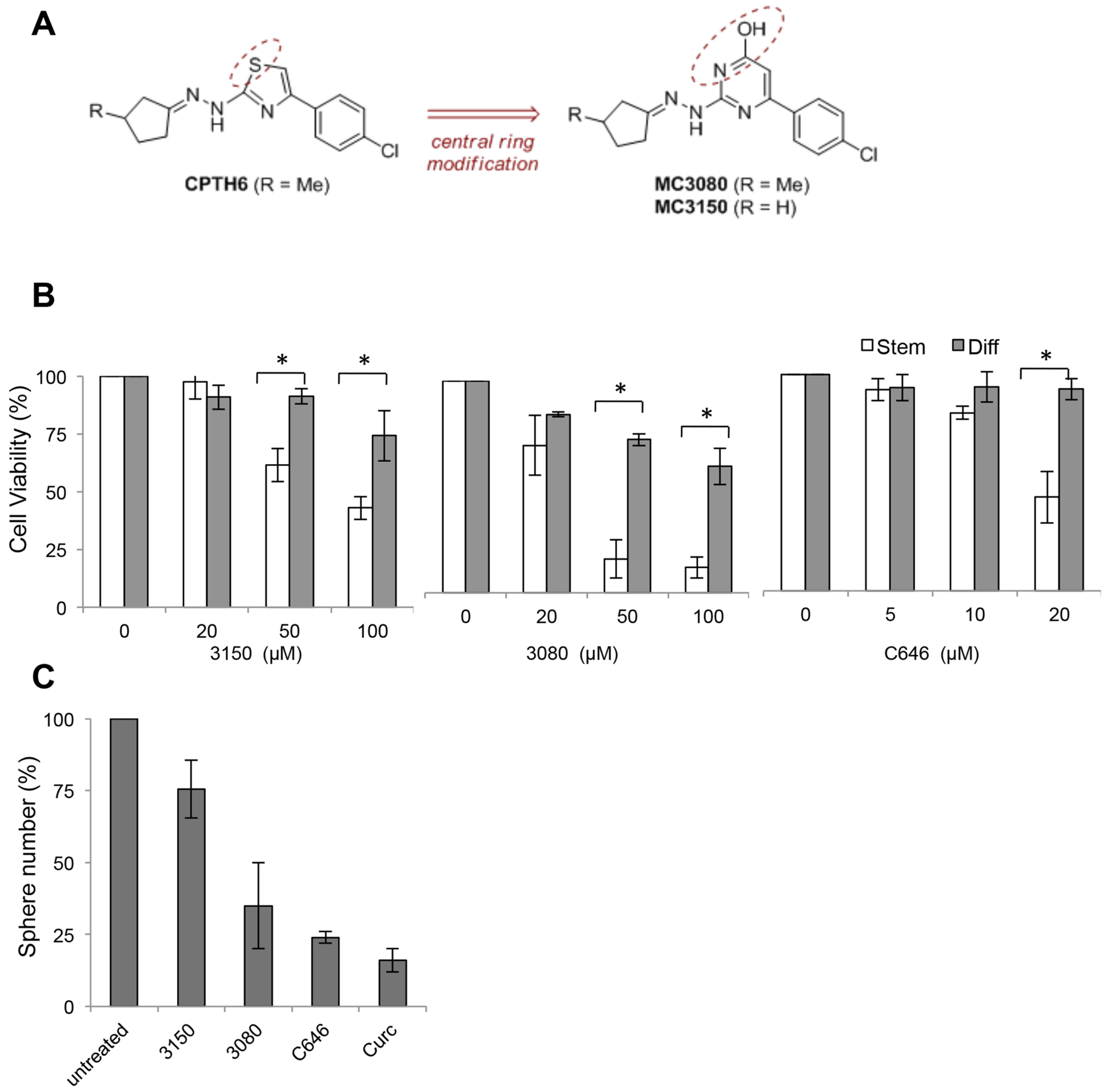

Supplementary Figure S4: HAT inhibition preferentially affects cell viability and self-renewal of LCSCs. A. Structural features of the two sets of HATi CPTH6 (left) and MC3080/3150 (right). The main structural difference regards the replacement of central thiazole nucleus of CPTH6 with a 4-oxopyrimidin-2-yl one in case of MC3080/3150. **B.** Analysis of cell viability by CellTiter-Glo assay in LCSC136 spheroids (Stem) and their differentiated progeny (Diff) exposed to increasing concentrations of 3080, 3150 and C646 for time ranging from 24h to 72h. The results are reported as "viability of drug-treated cells/viability of untreated cells" × 100 and represent the mean ± SD of three independent experiments. *$p<0.01$. **C.** Analysis of self renewal by tumorsphere formation of LCSC136 cells. Quantification of tumor sphere formation of LCSC136 cells treated with 3080 (50µM), 3150 (50µM), Curcumin (Curc, 40µM), C646 (30µM) at the indicated concentration. Data shown represent the percentage of spheres normalized to the number of seeded cells.

Supplementary Table S1: Characterization of primary culture derived from NSCLC surgical specimens

LCSC line	Tumor subtype	CSC-specific markers	Tumorigenicity
**136**	SSC	CD133 2,2%; ALDH low; CD44 99%	yes
**36**	SSC	CD133 90%; ALDH high; CD44 0.8%	yes
**18**	LCNEC	CD133 98%; ALDH high; CD44 0.1%	yes
**143**	AC	CD133 1,1%; ALDH low; CD44 74.8%	yes
**196**	SCC	CD133 1%; ALDH low; CD44 93%	yes
**223**	AC	CD133 1,7% ; ALDH high; CD44 62%	yes
**229**	AC	CD133 4%; ALDH low; CD44 96,2%	yes

Primary cultures were derived from NSCLC surgical specimens of different subtype (LCNEC, large cell neuroendocrine carcinoma; AC, adenocarcinoma; SCC, squamous cell carcinoma). Following tumor sample dissociation, cell suspensions were maintained in specific culture conditions that allowed the expansion of undifferentiated cancer stem cells (CSC), while negatively selecting serum- dependent tumor and accessory cells.

CSC-specific markers were analyzed by flow cytometry analysis and expressed as percentage of positive cells. ALDH expression and activity (defined as low or high) was evaluated by western blot and flow cytometry analyses. For tumorigenicity study, less than 10^5 cells of spheroid LCSC lines admixed with Matrigel were subcutaneously injected into NOD-SCID mice. The time of appearance of tumors, size and extent of growth was different for each LCSC line analyzed.
